# FRAX® tool, the WHO algorithm to predict osteoporotic fractures: the first analysis of its discriminative and predictive ability in the Spanish FRIDEX cohort

**DOI:** 10.1186/1471-2474-13-204

**Published:** 2012-10-22

**Authors:** Rafael Azagra, Genís Roca, Gloria Encabo, Amada Aguyé, Marta Zwart, Sílvia Güell, Núria Puchol, Emili Gene, Enrique Casado, Pilar Sancho, Silvia Solà, Pere Torán, Milagros Iglesias, Maria Carmen Gisbert, Francesc López-Expósito, Jesús Pujol-Salud, Yolanda Fernandez-Hermida, Ana Puente, Mireia Rosàs, Vicente Bou, Juan José Antón, Gustavo Lansdberg, Juan Carlos Martín-Sánchez, Adolf Díez-Pérez, Daniel Prieto-Alhambra

**Affiliations:** 1 Department of Medicine, Universitat Autònoma de Barcelona (UAB), Psg Vall d’Hebrón 119-129, 08035, Barcelona, Spain; 2 Badia del Vallès Health Centre, Catalan Health Institute. USR-MN-IDIAP Jordi Gol. c/ Bética s/n, 08214, Barcelona, Badia del Vallès, Spain; 3 Doctorate Program, Department of Medicine, Universitat Autònoma de Barcelona (UAB). Psg Vall d’Hebrón 119–129, 08035, Barcelona, Spain; 4 Sant Llàtzer Health Centre, Sanitary Consortium of Terrassa. c/ de la Riba 62, 08221, Barcelona, Terrassa, Spain; 5 Nuclear Medicine Service, Vall d’Hebrón University Hospital. Psg Vall d’Hebrón 119–129, 08035, Barcelona, Spain; 6 Granollers Centre Health Centre, Catalan Health Institute. c/ Museu 19, 08400, Barcelona, Granollers, Spain; 7 Can Gibert del Plà-Girona-2 Health Centre, Catalan Health Institute. c/ Sant Sebastià 50, 17005, Girona, Spain; 8 Montcada i Reixach Health Centre, Catalan Health Institute. Psg de Jaume I s/n, 08110, Barcelona, Montcada i Reixac, Spain; 9 Emergency Department, Hospital de Sabadell, Sanitary Consortium of Parc Taulí, Universitat Autònoma de Barcelona. Parc Tauli s/n, 08208, Barcelona, Sabadell, Spain; 10 Rheumatology Department, Hospital de Sabadell, Sanitary Consortium of Parc Taulí, Universitat Autònoma de Barcelona. Parc Tauli s/n, 08208, Barcelona, Sabadell, Spain; 11 Corbera de Llobregat Health Centre, Catalan Health Institute. c/ Buenos Aires, 9, 08757, Barcelona, Corbera de Llobregat, Spain; 12 Emergency Department, University Hospital of Bellvitge, Catalan Health Institute. University of Barcelona. c/ de la Feixa Llarga s/n, 08907, Barcelona, L'Hospitalet de Llobregat, Spain; 13 Primary Health Research Support Unit Metropolitana Nord, Catalan Health Institute-IDIAP Jordi Gol. Rambla 227, 08223, Barcelona, Sabadell, Spain; 14 Cabrils Health Centre, Catalan Health Institute. c/ Cal Batalló 3, 08348, Cabrils, Barcelona, Spain; 15 Bon Pastor Health Centre. Health Institute. c/ Mollerussa s/n, 08030, Barcelona, Spain; 16 Balaguer Health Centre, Catalan Health Institute. Universitat de Lleida. c/ Àngel Guimerà, 24 25600, Lleida, Balaguer, Spain; 17 Botnar Research Centre, Nuffield Department of Orthopaedics, Rheumatology and Musculoskeletal Sciences, University of Oxford, Oxford, OX3 7LD, UK; 18 Taradell Health Centre, Catalan Health Institute, C. Passeig del Pujaló, 5, 08552, Barcelona, Taradell, Spain; 19 Sanllehy Health Centre. Catalan Health Institute. Av. Mare de Déu de Montserrat, 16–18, 08024, Barcelona, Spain; 20 Poble Sec 3B Health Centre, CAP Manso. Catalan Health Institute. c/ Manso, 19–27, 08015, Barcelona, Spain; 21 Universidade de José do Rosàrio Vellano. UNIFENAS, Belo Horizonte. Rua Libano - Bairro Itapoã 66, Belo Horizonte, 31710-030, Minas Gerais, Brasil; 22 Universitat Internacional de Catalunya (UIC), c/ Josep Trueta s/n 08195 Sant Cugat del Vallès, Barcelona, Spain; 23 Institut Municipal d'Investigacions Mèdiques (IMIM)-Parc de Salut Mar, URFOA, Internal Medicine, Universitat Autònoma de Barcelona. Psg Marítim 25, 08003, Barcelona, Spain

## Abstract

**Background:**

The WHO has recently published the FRAX® tool to determine the absolute risk of osteoporotic fracture at 10 years. This tool has not yet been validated in Spain.

**Methods/design:**

A prospective observational study was undertaken in women in the FRIDEX cohort (Barcelona) not receiving bone active drugs at baseline. Baseline measurements: known risk factors including those of FRAX® and a DXA. Follow up data on self-reported incident major fractures (hip, spine, humerus and wrist) and verified against patient records. The calculation of absolute risk of major fracture and hip fracture was by FRAX® website. This work follows the guidelines of the STROBE initiative for cohort studies. The discriminative capacity of FRAX® was analyzed by the Area Under Curve (AUC), Receiver Operating Characteristics (ROC) and the Hosmer-Lemeshow goodness-of-fit test. The predictive capacity was determined using the ratio of observed fractures/expected fractures by FRAX® (ObsFx/ExpFx).

**Results:**

The study subjects were 770 women from 40 to 90 years of age in the FRIDEX cohort. The mean age was 56.8 ± 8 years. The fractures were determined by structured telephone questionnaire and subsequent testing in medical records at 10 years. Sixty-five (8.4%) women presented major fractures (17 hip fractures). Women with fractures were older, had more previous fractures, more cases of rheumatoid arthritis and also more osteoporosis on the baseline DXA. The AUC ROC of FRAX® for major fracture without bone mineral density (BMD) was 0.693 (CI 95%; 0.622-0.763), with T-score of femoral neck (FN) 0.716 (CI 95%; 0.646-0.786), being 0.888 (CI 95%; 0.824-0.952) and 0.849 (CI 95%; 0.737-0.962), respectively for hip fracture. In the model with BMD alone was 0.661 (CI 95%; 0.583-0.739) and 0.779 (CI 95%; 0.631-0.929). In the model with age alone was 0.668 (CI 95%; 0.603-0.733) and 0.882 (CI 95%; 0.832-0.936). In both cases there are not significant differences against FRAX® model. The overall predictive value for major fracture by ObsFx/ExpFx ratio was 2.4 and 2.8 for hip fracture without BMD. With BMD was 2.2 and 2.3 respectively. Sensitivity of the four was always less than 50%. The Hosmer-Lemeshow test showed a good correlation only after calibration with ObsFx/ExpFx ratio.

**Conclusions:**

The current version of FRAX® for Spanish women without BMD analzsed by the AUC ROC demonstrate a poor discriminative capacity to predict major fractures but a good discriminative capacity for hip fractures. Its predictive capacity does not adjust well because leading to underdiagnosis for both predictions major and hip fractures. Simple models based only on age or BMD alone similarly predicted that more complex FRAX® models.

## Background

The major manifestation or clinical consequence of osteoporosis is the appearance of osteoporotic fracture or fragility fracture [1]. It is well known that osteoporotic fractures involve a higher incidence of new fractures and lead to disability [2]. Hip fractures and those of the vertebrae with clinical manifestations are especially important since they carry an increase in mortality [3,4]. There is currently wide consensus regarding the need to develop strategies for the prevention of fractures and in the last years it has been recommended that the decision and the threshold of intervention be based on clinical assessment of risk of fragility fracture [5-8] and not only on the values of BMD and the relative risk as in the meta-analysis by Marshall D et al. [9].

Multiple epidemiological studies have described different clinical risk factors of osteoporotic fracture (CRFs) and which are been associated with an increased risk of developing osteoporosis and/or fragility fractures. Nonetheless, not all have determined a strong association, and the presence of these CRFs has not been uniform in the different studies and systematic reviews [10-14].

Most of the most powerful CRFs are concordant in different populations and, in general, similar for different fractures. Fractures related to falls have additional risk factors such as the number of falls, scarce physical activity and others such as the use of a walking stick, the need for help to get up from a chair, etc.). The CRFs associated with lifestyle such as smoking, alcohol intake or caffeine, low calcium consumption and scarce physical exercise have shown greater variability and lesser uniformity among the different studies [6,7]. Finally, the influence of some risk factors on the risk of fragility fracture has been demonstrated in different meta-analyses and systematic reviews [15-20]. As previously commented, since more than 15 years ago there has been evidence that BMD below the standard values is one of the important risk factors for fragility fracture [21,22]. More recently, however, other CRFs with as great or greater specific weight in the determination of risk of fragility fracture have been reported [11-13,22].

It is well known that there is an important variation in the relative risk of hip fracture in both men and women at an international level. The WHO itself has performed numerous investigations on this difference. In one of the latest studies this difference was defined as a standardised rate at 10 years, being, in the most extreme cases, 15-fold greater between countries such as Norway and Chile [23].

The studies performed by the Bone and Mineral Research Program, in Garvan Institute of Medical Research show that the combination of BMD and non-invasive clinical risk factors in a nomogram could be useful for identifying high-risk individuals for intervention to reduce the risk of hip fracture [24]. With the objective to make a purpose of when were the better moment and the patient who better benefits of new drugs available for the prevention of osteoporotic, World Health Organization Collaborating Centre for Metabolic Bone Diseases, University of Sheffield, UK developed the FRAX tool. Both are useful tools to estimate absolute risk of fracture for clinical practice but both have limitations: They discriminative ability was only moderate in older women (mean 74 years old) which may limit their clinical utility [25].

Both Garvan and FRAX are widely available tools: http://www.garvan.org.au/bone-fracture-risk/ and http://www.shef.ac.uk/FRAX/ but both models still need to be validated in different populations before they can be generalized to other populations and further studies will be needed to validate their contribution in selecting patients who will achieve fracture risk reduction with anti-osteoporosis therapy. With the current available algorithms, a possible clinical application may be to use FRAX as the primary model and to consider using Garvan in patients with recurrent fractures and falls [25].

Since the technical reports of 1994 [26] and their review in 2001 few changes have been made with respect to the WHO recommendations on the management of osteoporosis. In 2007, the WHO published a new tool for the evaluation of absolute risk of fragility fracture: the FRAX tool [27-30]. This tool was developed by WHO to evaluate fragility fracture risk for a 10 year period in patients for many countries [31-33].

The extension of a method for calculating the risk (probability) of fractures using the FRAX tool is foreseeable in Spain similar to what is occurring in other countries since its publication [34,35]. But before its clinical use its necessary to validate the calculator in a local cohort [29,30].

### Objectives

The objective of this study was to evaluate the discriminative and predictive capacity of the FRAX tool to determine osteoporotic or fragility fracture in Spain at 10 years.

This study describes the discriminatory capacity using the AUC-ROC *of the* FRAX tool to determine which Spanish women will have an osteoporotic fracture over the 10 years following the determination of the risk. On the other hand, the global predictive capacity of the FRAX tool has been calculated to detect the osteoporotic fractures on comparing the fractures observed over the 10 years with those expected by the FRAX tool.

## Methods

### Methods/design

The protocol, procedures and main characteristics of the study have recently been published [35].

Briefly, the FRIDEX cohort (**F**racture **RI**sk **f**actors and bone **DE**nsitometry type central dual **X**-ray) is constituted of men and women referred by general practitioners and specialists for undergoing central bone densitometry by Dual-energy X-ray absorptiometry (DXA) for the initial study of osteoporosis or treatment follow up, who accept to answer an extensive questionnaire on risk factors (QRF) for osteoporotic fracture (family history of osteoporosis and hip fracture, clinical risk factors and lifestyle habits related to diet and toxic substances) [35]. This cohort was started in 1999 at the Bone Densitometry Unit of the Department of Nuclear Medicine of the University Hospital Vall d’Hebrón, Barcelona, Spain.

During the baseline visit at the reference centre informed consent to participate was requested and a QRF for osteoporotic fractures is given during the visit and anthropometric parameters are determined. Ten years after the first QRF and DXA the patients were asked to answer a phone survey to know the evolution of the study variables and outcomes such as new personal or parental fractures, new disease or prescriptions.

### Study population and enrolment procedures

This multicentre study was carried out by family practitioners and other specialists who refer patients to the same reference centre for undertaking BMD. The criteria for referral followed the recommendations of the WHO of not performing a population screening but to select cases among those at greatest risk of having osteoporosis and subsequent osteoporotic fractures or the follow up and control of patients already receiving specific treatment.

Participants reside mostly in urban areas and were referred for DXA scan by family doctors, ambulatory specialists and hospital specialists.

Randomised sample (simple computerised randomisation stratified by sex) was obtained of women from 40 to 90 years of age in the FRIDEX cohort for 10 years since the baseline DXA and QRF.

### Eligibility criteria

#### Patient inclusion criteria

The study subjects were Caucasian women, ≥ 40 and ≤ 90 years of age at the time of inclusion in the FRIDEX cohort [35], understood and spoke the Spanish language, were able to respond to the initial questionnaire done at the surgery and a ten-year follow up structured telephone questionnaire (TQ). All accepted to participate in the study providing the corresponding verbal consent. Physically or psychically handicapped patients were included if the relatives or care providers accepted to answer the TQ.

### Patient exclusion criteria

Subjects < 40 or > 90 years of age at the time of the first DXA and QRF were excluded since the FRAX tool does not allow the calculation of the adjusted risk outside this age range. Patients with physical or psychological limitations impeding their participation and whose relatives did not accept to respond to the TQ were excluded as were those with Paget’s disease, cancer with bone involvement or disease which may simulate osteoporosis (i.e. myeloma). Patients from ethnic groups other than Caucasian were not included since other studies have demonstrated different risk characteristics. Patients not providing consent to the TQ and those without a telephone to contact or did not respond after 3 calls made at different times according to the procedure manual were also excluded from the study. Dead patients were not studied because of the impossibility of obtain all the study variables or to answer the questionnaire by relatives.

### Data collection

The sample ordering was performed using randomised numbers for each month and the calls were made in this order. The baseline variables of QRF and BMD were collected from January to July 2000. The follow up variables were collected at the same month during 2010 by TQ to complete the 10 years of follow up. The TQ was collected regarding the fragility fractures occurring from the time of inclusion until the date of the TQ as well as other information on known factors of fracture risk and falls. In all cases of fracture the medical records of the patients were reviewed and, when necessary, we requested a medical report for its validation. All cases of fracture that could not be verified or those arising from a motor vehicle accident or major trauma were excluded from analysis. Dead patients were not studied because of the impossibility of obtain all the study variables and to answer the questionnaire by relatives.

### Baseline variables

Height, weight, body mass index were obtained during baseline DXA scan. The rest of baseline items were obtained by semi structured questionnaire by interviewer during the same visit. On the other hand, the variables are set according to the instructions of the official website of FRAX [ http://www.shef.ac.uk/FRAX/tool.jsp?lang=sp]. The variables which are mentioned in the questionnaire were defined as well according to standard units of measurement for each. Regarding the risk of alcohol consumption, the quantification of consumption in standard drinks (UBEs) allows rapid quantification of consumption and its easy conversion into grams of pure alcohol. The value of the UBE in Spain with a slight North–south gap is set to 10 g of alcohol and is equivalent to a consumption of wine (100ml), sparkling wine (100 ml) or beer (200 ml) half and consumption of distilled or combined (25 ml). Weekly risky drinking for women and over 65 years is that is> 17 UBEs and men> 28-UBEs. The phone records of alcohol consumption have shown good validity and correlation in Mediterranean countries where alcohol consumption is widespread. Only in case of personal circumstances (deafness, slurred speech, etc.) a part of the information was obtained through regular cohabiting relatives of patients in 15 of 770 cases (1.9%). BMD measurement was determined by central DXA according to the 2007 recommendations of the International Society for Clinical Densitometry (ISCD) (available at: http://www.iscd.org/Visitors/positions/OfficialPositionsText.cfm) for the interpretation of the results using a Lunar GE model *Prodigy Advance* densitometer with 11.4 software and with BMD and T-score determination with NHANES III references. The densitometry diagnostic criteria used were the 1994 WHO criteria which classify the results into 3 groups according to the levels of BMD values of the femoral neck: normal (T-score >−1), osteopenia (T-score between −2.4 and −1 inclusive) and osteoporosis (T-score ≤ −2.5).

The estimated absolute risk of fracture during the 10-year period according to the FRAX tool was determined through the official website (version 3.2 accessed on October 2010). The calculations of the probability of fracture with or without the T-score of femoral neck and lumbar spine (L1-L4) were analysed in parallel by two blind investigators (patient entities were kept anonymous and were assigned an alphanumeric code). Two other blinded investigators reviewed the results and recalculated the data on the appearance of any difference.

### Analysis plan

The hip fractures during the follow up period were taken as the endpoint event. At first, all fractures were collected by TQ (structured interview), but were only included in the analysis if these fractures were verified against patients records. The characteristics of the population were described according to descriptive univariate analysis. We used the Chi-square test to evaluate the association between qualitative variables. The Student’s t-test or, if necessary, its non parametric equivalent, the Mann–Whitney U test, was implemented to evaluate the differences in the distribution of a quantitative variable according to the categories defined by a binary exposure. To assess the differences in the distribution of a quantitative variable according to the categories defined by a categorical variable with more than two categories, ANOVA analysis of variance or its corresponding non parametric test (Kruskal-Wallis) were used. The relative risk (RR) was calculated by quotient between prevalence of each risk factor in fractured women and in non-fractured.

To know the discriminating ability of the FRAX tool we used AUC-ROC and the Hosmer-Lemeshow goodness-of-fit test. The overall predictive capacity ratio was calculated by comparison of observed fractures (ObsFx) in the cohort and period and the expected fractures (ExpFx) by the FRAX tool [sum of individual probability of fracture from all women included/100].

The proportion of fractures expected is calculated by the sum of an individual probability of fracture from all women included/100. Model calibration is done by multiplying the FRAX result by the ratio ObsFx/ExpFx.

All the statistical tests were undertaken with a confidence interval of 95% and with the use of the 17th version of the SPSS statistical package.

This work follows the guidelines of the STROBE initiative for epidemiological studies [ http://www.strobe-statement.org/index.php?id=strobe-publications].

### Ethics

Procedures for human subject protection and the original protocol [35] were approved by the Clinical Research Ethics Committee of the Vall d'Hebron University Hospital, Barcelona, Spain and by the Ethical Committee of the Institut Universitari d’Investigació en Atenció Primària (IDIAP) Jordi Gol. Barcelona. Spain. Informed consent was obtained before beginning the interviews of all the patients.

## Results

**Figure 1:**
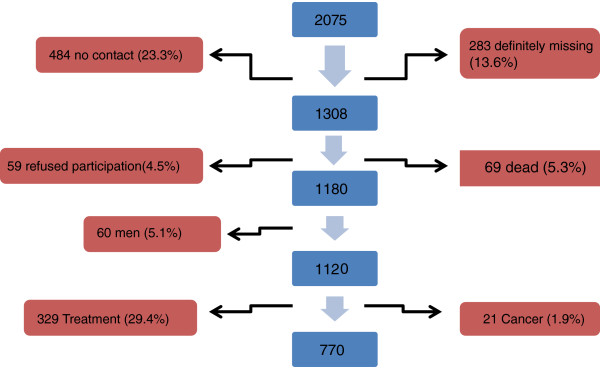
**Flow chart study.** Participant selection

Among the person completing 10 years since their inclusion in the cohort, 1,308 could be contacted for this study (Figure 1). About 69 (5.3%) patients died (43.4%). Thirty nine have been detected by searching the telephone number and detect the death. In the other 30 cases were detected through contact with family and reported only 2 cases of fracture between baseline and the date of death. A total of 770 women fulfilled the inclusion criteria and provided informed consent to participate.

During the 10 years of study 65 women presented a total of 82 major osteoporotic fractures which could be contrasted: 17 women with 18 hip fractures, 10 with 18 proximal humeral fractures, 25 with 30 forearm fractures, and 14 with 16 vertebral fractures. All the fractures were caused by low intensity impact according to the classical definition of fragility fracture [26].

Table 1 shows the baseline characteristics of the participants and those selected but did not participate in the study. No significant differences were observed between these two groups except that the participants ware one year younger on average (56.8 vs. 57.8 years) and the participants were taking glucocorticoids (3.7 vs. 5.9%).

**Table 1:** Risk factors among participants/non participants in the current study of FRIDEX cohort

	**Participants**	**Non Participants**	**p-value**
	**(n= 1180)**	**(n= 895)**	
Age years, mean (SD)	56.8 (8.0)	57.8 (8.5)	<0.001
Weight in Kg., mean (SD)	66.6 (11.5)	65.9 (11.1)	ns
Height in cm., mean (SD)	155.2 (5.9)	155.6 (6.0)	ns
BMI in Kg/cm2 , mean SD)	27.7 (4.7)	27.9 (4.2)	ns
Smoker, n (%)	132 (11.2%)	103 (11.5%)	ns
Alcohol Risk, n (%)	6 (0.5%)	5 (0.5%)	ns
Previous Fracture, n (%)	269 (22.8%)	245 (27.4%)	ns
Parental osteoporosis or fractures, n (%)	185 (15.7%)	138 (15.4%)	ns
Glucocorticoids, n (%)	44 (3.7%)	53 (5.9%)	0.024
Rheumatoid Arthritis, n (%)	12 (1.0%)	11 (1.2%)	ns
Calcium/Vit. D Supplements, n (%)	221 (18.7%)	187 (20.9%)	ns
Active Bone Drugs (antiosteoporotic drugs), n (%)	329 (27.9%)	277 (30.9%)	ns

Table 2 describes the main characteristics of the 770 participating women as well as the results of the variables or risk factors included in the FRAX tool and the results of the baseline DXA expressed as the result stratified according to the WHO classification. It also includes the variable of *falls in the previous year* which was assessed at the end of the study. The CRFs showing significant differences between women with fractures and those without fractures are: age, previous fractures, having rheumatoid arthritis and having a diagnosis of osteoporosis on DXA. The relative risks (RR) of the different CRFs are shown separately for major fracture and hip fracture in Tables 2 and 3.

**Table 2:** Baseline risk factors and falls in previous year for major fracture

	**65 Women with fracture**	**705 Women without fracture**	**P- value**	**CI 95%**	**RR**	**CI 95% RR**
Age (SD)	61.2 (9.7)	56.4 (7.7)	<0.001	2.4-7.3	2.62 (*)	(1.63 – 4.21)
Weight (SD)	67.4 (11.5)	66.6 (11.4)	0.559	ns	-	-
Height (SD)	155.3 (6.1)	155.1 (5.8)	0.805	ns	-	-
BMI (SD)	28.0 (4.4)	27.7 (4.7)	0.653	ns	1.19 (**)	(0.31 – 4.53)
Previous Fracture (%)	43.1	18.6	<0.001	12.1-36.9	2.91	(1.84 – 4.60)
Parental Hip Fracture (%)	15.4	15.6	0.963	ns	0.98	(0.52 – 1.88)
Smoker (%)	9.2	11.3	0.604	ns	0.81	(0.36 – 1.82)
Alcohol Risk (%)	1.5	1	0.508	ns	1.49	(0.23 – 9.45)
Glucocorticoids (%)	7.7	2.8	0.052	ns	1.15	(0.94 – 1.40)
Rheumatoid Arthritis (%)	4.6	0.7	0.024	1.2-9.0	4.61	(1.83 – 11.63)
Falls in previous year (%)	32.3	22.3	0.066	ns	1.59	(0.97 – 2.60)
Osteoporosis (baseline DXA) (%)	50.8	25.8	<0.001	12.4-37.6	4.96 (‡)	(1.98 – 12.43)
Osteopenia (baseline DXA) (%)	41.5	50.9	0.147	ns	2.36 (Φ)	(0.93 – 6.03)
Normal (baseline DXA) (%)	7.7	23.3	0.004	8.4-22.8	-	-

RR: Relative Risk.

(*) < 65 vs. ≥ 65 years.

(**) < 20 vs. ≥ 20.

(‡) Osteoporosis vs. normal.

(Φ) Osteopenia vs. normal.

**Table 3:** Baseline risk factors and falls previous year for hip fracture

	**17 Women with fracture**	**753 Women without fracture**	**P- value**	**CI 95%**	**RR**	**CI 95% RR**
Age (SD)	69.4 (7.1)	56.5 (7.8)	<0.001	9.2-16.7	11.49 (*)	(4.12 – 32.08)
Weight (SD)	64.8 (8.1)	66.7 (11.5)	0.498	ns	-	-
Height (SD)	153.1 (7.3)	155.2 (5.8)	0.139	ns	-	-
BMI (SD)	27.7 (3.0)	27.7 (4.7)	0.945	ns	1.02 (**)	(0.06 – 16.43)
Previous Fracture (%)	47.1	20.1	0.012	3.1- 50.9	3.42	(1.34 – 8.71)
Parental Hip Fracture (%)	17.6	15.5	0.738	ns	1.16	(0.34 – 3.98)
Smoker (%)	0.0	11.4	0.242	ns	0.225	(0.01 – 3.71)
Alcohol Risk (%)	0.0	1.1	1.000	ns	2.42	(0.16 – 37.26)
Glucocorticoids (%)	11.8	3.1	0.102	ns	3.97	(0.96 – 16.45)
Rheumatoid Arthritis (%)	11.8	0.8	0.012	4.3-26.3	12.7	(3.46 - 46.63)
Falls in previous year (%)	35.3	22.8	0.246	ns	1.81	(0.68 – 4.84)
Osteoporosis (baseline DXA) (%)	58.8	27.2	0.004	8.0-55.2	7.86 (‡)	(1.02 – 60.80)
Osteopenia (baseline DXA) (%)	35.3	50.5	0.215	ns	2.63 (Φ)	(0.32 – 21.65)
Normal (baseline DXA) (%)	5.9	22.3	0.106	ns	-	-

RR: Relative Risk, ns: no statistical significance.

(*) < 65 vs. ≥ 65 years.

(**) < 20 vs. ≥ 20.

(‡) Osteoporosis vs. normal.

(Φ) Osteopenia vs. normal.

**Figure 2:**
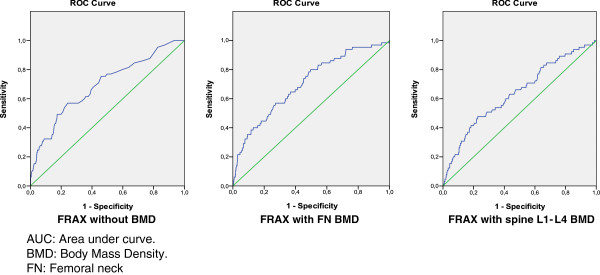
AUC ROC of FRAX tool for major fracture in FRIDEX cohort

**Figure 3:**
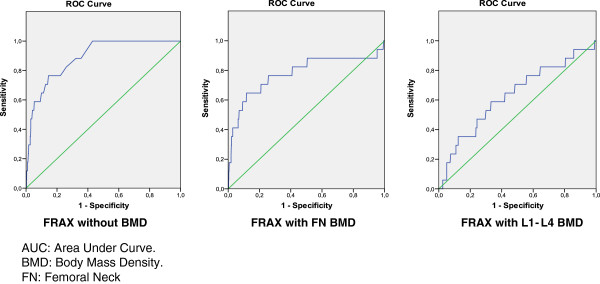
AUC ROC of FRAX tool for hip fracture in FRIDEX cohort

The values of the different AUC-ROC for major and hip fracture calculated in the cohort of Spanish women are shown in Table 4. That is, of BMD by DXA with the T-score of the femoral neck (FN) and with the T-score of spine L1-L4 and the FRAX tool in three ways: without BMD, with the FN T-score and with spine L1-L4 T-score. The best result was for FRAX tool for hip fracture without the T-score (0.888). In all cases the results presented significant differences with the reference (0.50) except for BMD with spine L1-L4 T-score (p=0.067). Figures 2 and 3 are graphs of the AUC-ROC of the FRAX tool for major fracture and hip fracture. A determination of the AUC-ROC specifically for vertebral fracture was performed, being 0.752 (CI 95%; 0.643-0.861) for the FRAX tool without BMD, 0.815 (CI 95%; 0.725-0.905) with the FN T-score and 0.710 (CI 95%; 0.575-0.844) with L1-L4 T-score, without significant differences among them (p=0.157) (graph not shown). We compare AUC of the ROC curve of FRAX tool for major and hip fracture with a simple model including only age. The AUC in a model that includes only age was 0.668 for major fracture and 0.882 for hip fracture with no significant differences with the results of FRAX tool (p=0.565 and p=0.976 respectively).

**Table 4:** Area Under Curve (AUC) of Receiver Operating Characteristics (ROC)

		**AUC ROC**	**CI 95%**	**p - value**
AUC ROC 10-year prediction of MAJOR FRACTURE	BMD with FN T-score	0.661	(0.583-0.739)	p<0.001
	BMD with L1-L4 T-score	0.638	(0.565-0.711)	p<0.001
	FRAX® tool without BMD	0.693	(0.622-0.763)	p<0.001
	FRAX® tool with FN T-score	0.716	(0.646-0.786)	p<0.001
	FRAX® tool with spine L1-L4 T-score	0.712	(0.644-0.780)	p<0.001
AUC ROC 10-year prediction of HIP FRACTURE	BMD with FN T-score	0.779	(0.631-0.929)	p<0.001
	BMD with L1-L4 T-score	0.630	(0.487-0.773)	(ns)
	FRAX® tool without BMD	0.888	(0.824-0.952)	p<0.001
	FRAX® tool with FN T-score	0.849	(0.737-0.962)	p<0.001
	FRAX® tool with spine L1-L4 T-score	0.767	(0.658-0.876)	p<0.001

AUC ROC: Area Under Curve of Receiver Operating Characteristics; BMD: Bone Mineral Density; FN: Femoral Neck; L1-L4: Lumbar Spine; CI: Confidence Interval; ns: Not significant.

Around 27 major fractures and 6 hip fractures were expected with the FRAX tool without BMD, while around 30 major fractures and 7 hip fractures were expected with the inclusion of the T-score of the femoral neck in the FRAX tool (Table 5).

**Table 5:** Ratio of Observed fractures/Expected fractures by FRAX tool

	**MAJOR FRACTURES**				**HIP FRACTURES**			
	**Obs Fx**	**Exp Fx**	**Ratio Fx Obs/Exp**	**CI 95%**	**Obs Fx**	**Exp Fx**	**Ratio Fx Obs/Exp**	**CI 95%**
FRAX without BMD	65	26.7	2.4	(1.9 - 3.1)	17	6.0	2.8	(1.7- 4.6)
FRAX with FN T-score	65	29.9	2.2	(1.7 - 2.8)	17	7.3	2.3	(1.4 - 3.8)

MAJOR FRACTURES (hip, vertebra, humerus, wrist). Fx: Fracture.

ObsFx: Observed fractures; ExpFx: Expected fractures; CI: Confidence Interval;

BMD: Bone Mineral Density; FN: Femoral Neck.

The ObsFx/ExpFx ratio was 2.4 (CI 95%; 1.9 - 3.1) for major fracture and 2.8 (CI 95%; 1.7 - 4.6) for hip fracture (Table 5) with the FRAX tool without BMD and 2.2 (CI 95%; 1.7 - 2.8) and 2.3 (CI 95%; 1.4 - 3.8), respectively with femoral neck T-score. Expressed in percentages, the FRAX tool without BMD predicts 41.1% of the cases of women with major fracture in 10 years and 46% on adding the algorithm of the T-score of the femoral neck, with these values being 35.5% and 42.8% for hip fractures, respectively.

With respect to the analysis of the sample of the FRIDEX cohort we performed a goodness-of-fit test which stratifies the results in quintiles of risk associated with quintiles of results of fracture.

**Figure 4:**
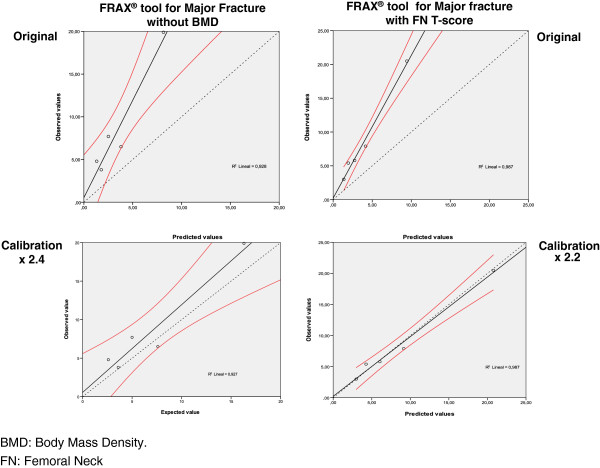
**Hosmer-Lemeshow test.** FRAX tool for Major Fracture

**Figure 5:**
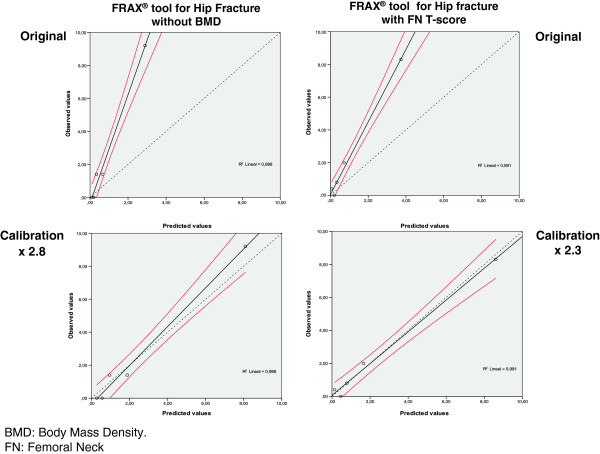
**Hosmer-Lemeshow test.** FRAX tool for Hip Fracture

Figure 4 shows the Hosmer-Lemeshow test for major fracture, with the cases of the sample distributed into quintiles and the line of regression for the results of the FRAX tool without BMD and with the FN T-score. The lower part of the figure represents the same results after calibration (simulation) by the number of times that the ObsFx is greater than the ExpFx (Table 5). Figure 5 shows the results for hip fracture in the same way.

## Discussion

According to the comparative analysis of the baseline characteristics between the participants and the non participants for any reason we found that the non participants did not differ from the participants except in that they were one year older and more patients were on glucocorticoids. Thus, the participants in the study did not present worst conditions of the cohort.

Self-reported generally even structured interview have a significant correlation with those in the medical record. In any case always been found documented as explained. In all cases of fracture the medical records of the patients were reviewed and, when necessary, we requested a medical report for its validation. All cases of fracture that could not be verified or those arising from a motor vehicle accident or major trauma were excluded from analysis, fractures in the history of the subjects under study. A potential limitation of self-reported fractures is in vertebral fractures. In our study the total self-reported fractures were 16% higher than they were registered and so were excluded from the final analysis. It can be an advantage for risk predictions proposed by FRAX.

The present study is centered on the discriminatory and predictive capacity of the FRAX. Analysis of the AUC-ROC was used to analyse the discriminatory capacity of this tool. As shown in (Table 4) the results of the FRAX without DXA values were greater than the AUC-ROC of BMD with values of the T-score of the femoral neck. Thus, these results demonstrate that the FRAX without the determination of BMD presents a discriminatory capacity not inferior to and even somewhat better than the DXA, according to the AUC-ROC. Analysis of the BMD with the DXA technique for the axial skeleton has traditionally been considered as the best predictive test known to determine fragility fractures [9,26,36] with the strategy of intervention for their prevention in medical practice having been based on this test in Spain [35] and in the remainder of the international scientific community until the appearance of the importance of other risk factors for fracture [27-33].

On analysing the role of the determination of BMD of L1-L4 in the different tests, it was found that the discriminatory capacity for major fracture using the AUC- ROC was lower than that of the determination of BMD with the T-score of the femoral neck, although statistical significance was maintained (Table 4). This inferiority was maintained for hip fracture but with no significant differences since the confidence interval integrates the value 0.50 which is the value of statistical significance for this test. Part of the debate on the possible weaknesses of the FRAX has been centered on the lack of the BMD values of the lumbar spine in its algorithm. This criticism is based on the traditional consideration that the BMD of each area presents the best predictive capacity for fractures in the same area, especially for the vertebrae and the hip [36] and, thus, it has been argued that the prediction of vertebral fractures could be improved. The discriminatory capacity measured with the AUC-ROC worsened with the incorporation of the L1-L4 T-score in the algorithm of the FRAX for major or hip fractures (Table 4). This result is congruent, but on introducing the L1-L4 T-score value in the FRAX (as a simulation) to analyse what would happen with vertebral fracture, the result of the AUC-ROC for vertebral fracture worsened slightly with respect to that obtained with the FN T-score, although without significant differences. Thus, on introducing the values of the L1-L4 T-score in the FRAX in this study the result did not provide an improvement in the discrimination of vertebral fractures measured with the AUC-ROC. Although it has described that a correction can adapt the lumbar spine BMD and improve the prediction for major and vertebral fractures of FRAX [31] in our study by incorporating the lumbar spine BMD did not improve the discriminative ability of FRAX measured by AUC with femoral neck BMD neither for major or vertebral fracture (data not showed).

The adjusted predictive capacity of the FRAX analysed using the ObsFx/ExpFx ratio was far from the 1 value which would be the desired result in the case of good adjustment of the predictive capacity of the FRAX in our country. In our cohort this ratio was of 2.4 for major fracture and 2.8 for hip fracture. These values improved minimally on the introduction of the T-score of the femoral neck in the algorithm (2.2 and 2.3 respectively). Indeed, the FRAX predicted the risk of major fracture in 41.1% of the women and 35.5% for hip fracture without BMD, with these values improving only slightly with 46% and 42.8%, respectively on performing the BMD with DXA.

These data seem to coincide with the analysis recently carried out in two cohorts of French women with a similar overall discriminatory value for fracture and low overall sensitivity (48-50% for FRAX predictions) and better than BMD alone [33,37]. In Spain our group previously demonstrated that the FRAX has good capacity to detect densitometric osteoporosis but also with imbalance in the predictive capacity [38-40]. Nonetheless, a two recent studies in Spain had shown similar results to ours for major fractures with an ObsFx/ExpFx ratio of 3.1 (CI 95%: 2.8-3.5) and 0.8 (CI 95%: 0.7-1.1) for hip fracture [41]. Although the initial formation of the two cohorts followed very similar schemes, the method of follow up in our study was notably different. In the present study we only analysed fragility fractures reported by the women, which could be contrasted with electronic record or clinical reports. In the second study the results of ratio ObsFx/ExpFx were 0.66 and 1.10 for major and hip fracture respectively [34]. The most important methodological differences were that the study was carry out for a three years period, the authors do not included vertebral fractures [34].

The ROC curve has several problems. For analysis of sensitivity and specificity we have not a gold standard of FRAX for Spanish population. Moreover, ROC needs a gold standard of illness (fracture) and we do not have because of the electronic records are not completely reliable and we needed to make a double check (self-reported validate against records). On the other hand, the area under the ROC curve is important, since it measures the discrimination power of the model. Nevertheless, tests of discrimination alone are not sufficient for model evaluation, since they do not indicate whether calibration is also good [34,35,42].

In our study, on application of the Hosmer-Lemeshow test a good correlation was observed between the different quintiles of risk in all the simulation (Figures 4, 5) but with a line which groups the results of the regression deviated from the reference toward the values observed. This circumstance led us to carry out a calibration multiplying each of the values resulting from the prediction made by the FRAX by a constant based on the ObsFx/ExpFx ratio for major fracture and for hip fracture. As shown in the lower part (calibration) of Figures 4 and 5, on multiplication of the results of the FRAX by the ObsFx/ExpFx ratios, the results with their CI 95% adjust perfectly to the diagonal of reference in the Hosmer-Lemeshow test.

The FRAX tool can therefore be considered to present with a poor discriminatory capacity for women to have major osteoporotic fractures within 10 years, with this capacity being good for hip fractures without the need of determining the BMD, although this improves somewhat with its determination. The FRAX tool shows a scarce predictive capacity of the risk of fracture and predicts less than 50% of those which occur. The reason for this underdiagnosis may be because the Spanish cohort introduced as the reference in the FRAX tool is not representative of the current female population since these women present significantly more fractures than those actually predicted by the FRAX tool.

We have excluded from the analysis of the cohort of women receiving active treatment for the bone at baseline of the study because of the FRAX has so defined, but we have not been excluded women who received treatment during the 10-year period. This can be a potential confounding factor, however exclude women would mean removing the greatest potential for fracture, but keep going who have received treatment can be reduced the all risk of new fractures observed. Other potential confounding factor can be the Calcium/Vit D supplement intake because we have not excluded at baseline or during the study period. There is important discussion in the literature about the role of these supplements in reducing the risk of fracture, except in a subgroup of patients taking bone active drugs for the potential hypocalcaemia or in patients admitted to nursing homes. These patients are not included in this study. Moreover there is no significant difference between Calcium/Vit D supplement intake between participants and no participants.

New epidemiological studies are needed in our country to compare these results on major and other fragility fractures which, although not severe, also affect the quality of life [43]. However, together with other authors in our country [6,10,34,38-41] we believe that there are sufficient data to promote the habit of investigating the risk factors of fragility fracture among Spanish physicians, especially in primary care, to determine the absolute risk and be able to propose changes in lifestyle in persons with a high risk as well as evaluate which patients should be referred for determination of the BMD by DXA [38]. In our opinion, the current state of the FRAX needs some adjustments such as those proposed in this study. Something similar to this need for adaptation and adjustments happened in Spain with the application of the first Framingham-type cardiovascular risk scales which required adaptations such as the REGICOR scale and others in our country [44-46].

We know that the promoters of the FRAX are committed to the adaptation of the tool to the different countries with the publication of new studies such as what has been done up to now. We also consider that with improvements this may be a very useful tool especially in the first level of care and this has been demonstrated by the important extension in its use worldwide [28,35].

## Conclusions

In summary, FRAX without BMD demonstrates a poor discriminative capacity for major fractures and a good discriminative capacity for hip fractures with the AUC ROC for Spanish women but its predictive capacity does not adjust well with the current algorithm leading to underdiagnosis for major fracture and hip fractures. On introducing the values of the L1-L4 T-score in the FRAX tool, the result did not provide an improvement in the discrimination of vertebral fractures measured with the AUC-ROC. Simple models based on age or BMD alone predicted 10-year risk of major and hip osteoporotic fractures, as well as more complex FRAX models.

We advise our Spanish colleagues to use the FRAX tool in clinical practice but weighing the resulting value of each individual case of the FRAX without BMD by a calibration value to obtain an absolute risk value of major o hip fracture at 10 years. New studies may allow a single value which is easier to remember in clinical practice. The result obtained will be more adjusted to the reality of the risk of fragility fracture in our country according to the results found in the present and other studies [34,38,41].

### Study limitations and strengths

Our study has some strengths and limitations. We assumed that women in the FRIDEX cohort could have a higher risk of osteoporotic fractures than the general population because it is a population that had previously been selected to undergo a DXA scan for some reason. However it is important to know the profile of women who are selected to perform the DXA-scan by general practitioners and other specialists as may higher but close to the general population over 50 years. Fractures occurring in the participants were followed by an ad-hoc TQ taking into account the traditional low response rates by post in previous epidemiological studies conducted in Spanish population [36]. However, all fractures included were verified against patient records.

Other potential confounders and biases are that we excluded those who died during the follow-up, the collection of incident fractures is captured in retrospect, the validation records was only for patients with fractures and, as well, usually the electronic registers of fracture tends to be less records than actually occur. To minimize these potential biases we have verified all self-reported fractures and not included in the study which did not fulfill both (self-reported and recorded). Therefore, this type of analysis tends to benefit the predicted fractures in the ratio ObsFx/ExpFx.

We are aware that the authors of the FRAX tool apply only the DXA value of the femoral neck because of the absence of improvement in the prediction of major fracture risk with the use of the lumbar spine T-score. This has been one of the main criticisms related to the FRAX tool.

As strengths of the study, 4 investigators were involved in the operating systems to verify the calculations of the values of FRAX and all hip fractures included in the analysis were contrasted. The FRIDEX study is a prospective population-based cohort study, being one of the first studies to follow Spanish women over a 10-year period to determine the incidence of fragility fractures.

## Abbreviations

AUC: Area Under Curve; BMD: Bone Mineral Density; CRFs: Clinical risk factors; DXA: Dual-energy X-ray Absorptiometry; FN: Femoral neck; FRAX® and FRAX: Fracture Risk Assessment Tool; FRIDEX: Fracture Risk Factors and Bone Mass Density by DXA cohort; ISCD: International Society for Clinical Densitometry; NAHNES: National Health and Nutrition Examination Survey; NOGG: National Osteoporosis Guideline Group; ObsFx/ExpFx: Observed fractures/Expected fractures ratio; QRF: Questionnaire of Risk Factors; RF: Risk Factor; RR: Relative Risk; ROC: Receiver Operating Characteristics; REGICOR: Registre Gironí del Cor [ http://www.regicor.org]; TQ: Telephone Questionnaire 10-year follow up; WHO: World Health Organization.

## Competing interests

The authors declare that they have no competing interests.

## Authors' contributions

RA is the principal investigator, project design and direction, preparation and review of the manuscript. GR coordination of field work, preparation and review of the manuscript. GE coordination and management of the cohort, review of the manuscript. AA coordination and analysis of the FRAX values, review of the manuscript. MI, NP, MZ, SG, PS, SS, VB, MR, MCG, FL-E, GL, JJA, GPL, JP-S and AP field work, calculation of the FRAX values and review of the manuscript. JCM statistical analysis and management of the database, review of the manuscript. EG, EC, YF, PT, DP-A and AD-P scientific support and methodological expert, review of the manuscript. All authors have read and approved the final manuscript.

## Pre-publication history

The pre-publication history for this paper can be accessed here:

http://www.biomedcentral.com/1471-2474/13/204/prepub
